# Annual temperature, body size, and sexual size dimorphism in the evolution of Pyrgomorphidae

**DOI:** 10.1002/ece3.70188

**Published:** 2024-08-21

**Authors:** Raúl Cueva del Castillo, Salomón Sanabria‐Urbán, Ricardo Mariño‐Pérez, Hojun Song

**Affiliations:** ^1^ Universidad Nacional Autónoma de México; Facultad de Estudios Superiores Iztacala Tlalnepantla Mexico; ^2^ University of Michigan Ann Arbor Michigan USA; ^3^ Texas A&M University College Station Texas USA

**Keywords:** body size, grasshoppers, sexual dimorphism, weather

## Abstract

In many animal species, larger body size is positively correlated with male mating success and female fecundity. However, in the case of insects, in high seasonality environments, natural selection favors a faster maturation that decreases the risk of pre‐reproductive death. However, this advantageous adaptation comes at a tradeoff, resulting in a reduction in body size. Maturation time is influenced by environmental factors, such as temperature, season length, and food availability during the rains. The geographic variation in these parameters provides an opportunity to study their impact on the adaptive evolution of body size in Pyrgomorphidae grasshoppers. These grasshoppers exhibit remarkable variation in body size and wing development and can be found in diverse plant communities across Africa, Asia, Australia, and tropical America. In this study, we utilized a phylogenetic approach to examine the evolution of body size, considering climatic factors, and the influence of sexual selection on size differences between males and females. We found a positive correlation between mean annual temperature and sexual size dimorphism (SSD). Remarkably, species exhibiting a strong bias toward larger females were found to be adapted to regions with higher temperatures. In the Pyrgomorphidae family, an intermediate body size was identified as the ancestral trait. Additionally, winged male and female grasshoppers were observed to be larger than their wingless counterparts. Despite the potential conflicting pressures on body size in males and females, these grasshoppers adhere to Rench's Rule, suggesting that sexual selection on males' body size may explain the evolution of SSD.

## Graphical abstract

In this study, using a phylogenetic comparative approach, we explored the impact of climatic conditions on the evolution of body size and sexual size dimorphism in pyrgomorph grasshoppers.

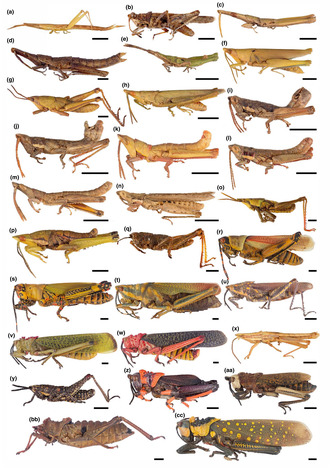

## 1 INTRODUCTION

In many animal species, a large body size increases male mating success (Andersson, 1994), while in females, it increases their fecundity (Honěk, 1993; Ridley, 1983). However, in insects, body size is closely linked to maturation time (Cueva del Castillo & Nuñez‐Farfán, 1999, 2002), which can be influenced by environmental factors, such as temperature, season length, and food availability (Blanckenhorn & Demont, 2004; Chown & Gaston, 2010; Sanabria‐Urbán et al., 2015). In temperate regions or high altitudes, the survivorship of insects declines sharply at low temperatures, at the end of the fall and in the winter (Amarasekare & Sifuentes, 2012), and natural selection favors shorter development times to reduce the risk of pre‐reproductive death, but this comes at the cost of smaller body size (Thornhill & Alcock, 1983; Dingle et al., 1990; Abrams et al., 1996; Ramírez‐Delgado & Cueva del Castillo, 2022). Conversely, in regions with lower latitudes and altitudes, where temperature variations are minimal and plant growth is abundant, resulting in an abundance of food for herbivorous insects (Amarasekare & Sifuentes, 2012; Yom‐Tov & Geffen, 2006), larger body sizes are favored by both natural and sexual selection in univoltine insect species (Kozlowski et al., 2004; Horne et al., 2015; Ramírez‐Delgado & Cueva del Castillo, 2022).

**FIGURE 1:**
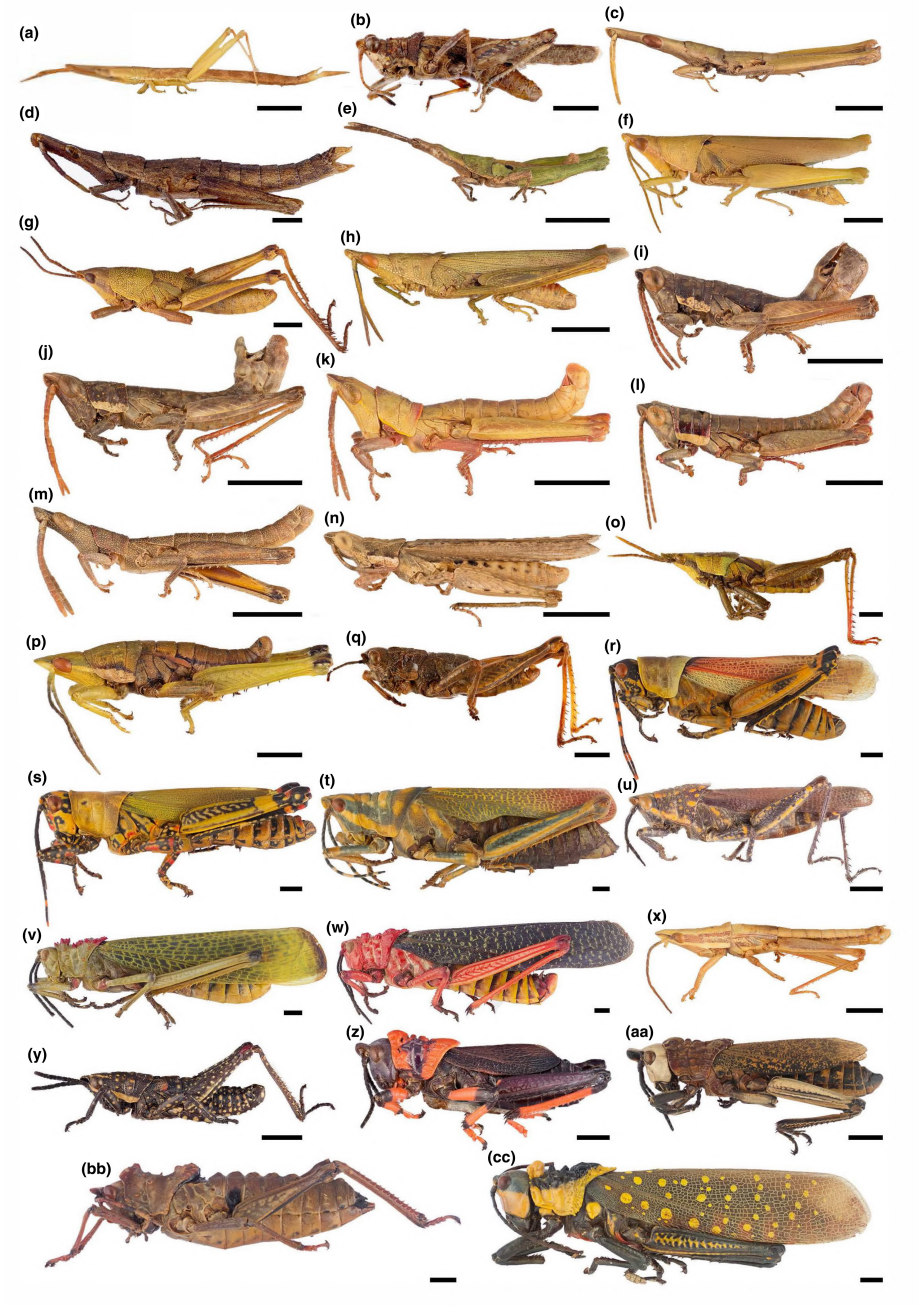
Pictures of most of the species of the family Pyrgomorphidae analyzed in this study: (a) *Psedna nana*; (b) *Chrotogonus hemipterus*; (c) *Omura congrua*; (d) *Algete brunneri*; (e) *Jaragua oviedensis*; (f) *Tagasta indica*; (g) *Yunnanites coriacea*; (h) *Atractomorpha aberrans*; (i) *Piscacris robertsi*; (j) *Ichthyotettix mexicanus*; (k) *Sphenotettix nobilis*; (l) *Pygotettix pueblensis*; (m) *Sphenacris crassicornis*; (n) *Pyrgomorpha conica*; (o) *Prosphena scudderi*; (p) *Sphenarium histrio*; (q) *Mekongiella kingdoni*; (r) *Zonocerus elegans*; (s) *Zonocerus variegatus*; (t) *Poekilocerus pictus*; (u) *Poekilocerus bufonius*; (v) *Phymateus viridipes*; (w) *Phymateus morbillosus*; (x) *Colemania sphenarioides*; (y) *Monistria concinna*; (z) *Dictyophorus spumans*; (aa) *Dictyophorus griseus*; (bb) *Parapetasia femorata*; (cc) *Aularches miliaris*. All males except d, f, t, & bb. Scale bar = 5 mm.

In this study, using a phylogenetic framework, we analyzed the trends in the evolution of the body size of grasshoppers in the family Pyrgomorphidae. These grasshoppers are among the most recognizable orthopteran lineages in the world. Several species are known for their vibrant color patterns, elaborate sculptures, and large sizes (Figure 1) (Zahid et al., 2021). Nonetheless, most pyrgomorph species are actually cryptically colored. There is a large variation in their sizes, and there are winged and wingless species The species of the family are distributed in Africa, Asia, and tropical America (Mariño‐Pérez & Song, 2019). Thus, they can be found in a wide variety of plant communities, including xerophytic, temperate, tropical deciduous, and rain forests. The variation in the niches they inhabit opens the opportunity to test the impact of the adaptive evolution on their body size.

We analyzed the evolutionary trends of body size among the pyrgomorphids, with special attention to the possible constraints imposed by the presence of wings. Furthermore, we explored the influence of climatic conditions on the evolutionary trajectory of both male and female body size. We also evaluated if the body size divergence in males and females can be attributed to sexual selection or fecundity selection testing the Rensch's rule. In several groups of animals, the magnitude of SSD increases with body size when males are larger than females, but it decreases with body size when females are larger than males (Rensch, 1950; Abouheif & Fairbairn, 1997; Fairbairn, 1997). This macro‐evolutionary pattern is known as Rensch's rule (Rensch, 1950) and it has been associated with mating systems with high levels of mate competition (Abouheif & Fairbairn, 1997; Fairbairn, 1997). The converse trend, where female size varies more than male size, is less common, but seems to be the result of strong fecundity selection acting on females (Blanckenhorn et al., 2007; Foellmer & Moya‐Larano, 2007; Webb & Freckleton, 2007).

Because environmental conditions can impact body size in grasshoppers, we expected that larger species of grasshoppers would be found in regions with low seasonality, high temperatures, and ample precipitation. These environmental factors contribute to the abundance of food for herbivorous insects, ultimately impacting their body size (Amarasekare & Sifuentes, 2012; Yom‐Tov & Geffen, 2006). In addition, because resource allocation also plays a role, as flight can reduce resources for egg production, resulting in a tradeoff between flight and fecundity (Tigreros & Davidowitz, 2019), we hypothesized that winged males and females would exhibit smaller body sizes compared with their wingless counterparts. We also conducted allometric regressions of male body traits on female traits to assess whether sexual selection on male body size has been stronger than fecundity selection on females. As a result of stronger directional sexual selection on male body size, according to the allometric relationship predicted by Rensch's rule, a greater evolutionary divergence in male size than in female size would be expected (Berner & Blanckenhorn, 2006; Fairbairn, 2007; Stillwell et al., 2010). Thus, the slope of the allometric regression of male size on female size should be steeper than 1 (Rensch 1950; Fairbairn, 1997).

## 2 MATERIALS AND METHODS

### 2.1 Morphological traits

**FIGURE 2:**
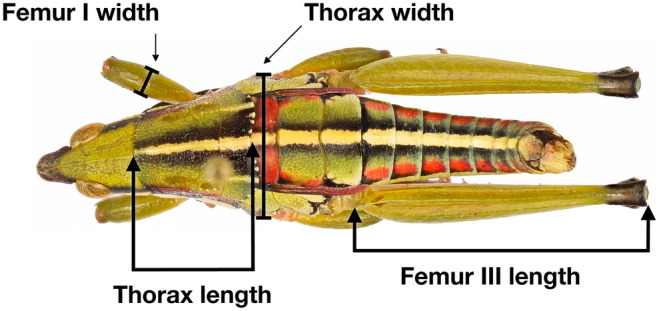
Anatomical characteristics measured in the grasshoppers studied species.

We measured museum specimens of taxa belonging to Pyrgomorphidae that we had previously collected and used to build morphological and molecular phylogenies deposited at the Texas A&M University Insect Collection (TAMUIC) (see Mariño‐Pérez & Song, 2018, 2019; Zahid et al., 2021), specimens deposited at the Academy of Natural Sciences of Drexel University in Philadelphia (ANSP), and at the University of Michigan Museum of Zoology Insect Collection (UMMZ), previously published *Sphenarium* data (Sanabria‐Urbán et al., 2015), and morphological data from other Mexican pyrgomorphids collected by Sanabria‐Urbán et al., 2015. Using a digital caliper (Mitutoyo Corp., Tokyo, Japan), we measured femur III length, width, and length of the thorax and femur I width of each adult male and female of each species (Figure 2). The length and width of the thorax and the length of the femur III summarize the variation in the body size of both sexes, whereas femur I is important in the competition between males for mating opportunities with females (Cueva del Castillo et al., 1999; Sanabria‐Urbán et al., 2015). Considering the total number of individuals for each taxon, we averaged the values of the four morphological traits per species and sex (Table 1). The values obtained were then log‐transformed prior to their utilization in the comparative analyses. Furthermore, we calculated the sexual size dimorphism index (SSDI) for each trait, following the methodology proposed by Lovich and Gibbons (1992) (Table 2). This index expresses SSD as [(length of larger sex/length of smaller sex) – 1]. For convention, the SSDi is arbitrarily changed to negative when males are the larger sex. Conversely, when females are the larger sex, the SSDi will be represented by a positive value.

**TABLE 1:** Morphometric measurements of males and females of 32 species of the family Pyrgomorphidae.

SPECIES	*n*	Males				*n*	Females			
		Mean F I	Mean F III	Mean TL	Mean TW		Mean F I	Mean F III	Mean TL	Mean TW
*Algete brunneri*	6	0.58	8.66	3.53	2.77	5	0.79	11.89	5.41	4.71
*Atractomorpha sinensis* ^a^	16	0.63	10.42	4.39	3.06	10	0.73	14.43	6.52	4.97
*Aularches miliaris* ^a^	12	1.95	21.03	12.63	10.62	13	1.79	21.00	13.73	11.86
*Chrotogonus hemipterus*	11	0.52	5.92	2.36	3.91	12	0.72	9.22	4.13	7.78
*Colemania sphenarioides*	2	0.98	10.23	4.14	3.36	2	0.89	12.78	6.21	4.41
*Dictyophorus griseus* ^a^	21	2.03	16.84	12.63	10.81	27	2.00	18.67	13.48	11.75
*Dictyophorus spumans* ^a^	15	2.01	20.21	12.76	10.87	21	2.26	23.13	15.88	13.82
*Ichthyotettix mexicanus*	13	0.80	8.37	3.46	2.60	11	0.84	10.76	5.64	3.94
*Jaragua oviedensis*	1	0.47	7.54	2.40	3.42	1	0.62	9.06	3.79	5.70
*Mekongiella kingdoni*	1	1.58	14.75	6.21	8.06	1	1.19	15.03	6.25	8.27
*Monistria consobrina*	1	1.39	11.21	5.66	5.17	1	1.55	15.57	9.57	9.66
*Monistria discrepans*	10	0.82	9.26	3.99	3.96	5	1.00	12.39	6.61	6.61
*Omura congrua*	20	0.73	12.34	4.28	3.50	19	0.90	16.74	6.74	6.91
*Parapetasia femorata*	10	1.39	14.07	10.06	8.67	10	1.70	20.92	14.7	13.96
*Phymateus morbillosus* ^a^	13	2.09	27.25	11.84	10.79	10	2.49	28.58	15.49	15.48
*Phymateus viridipes* ^a^	8	1.82	24.14	10.61	9.76	11	2.19	30.27	14.37	13.87
*Piscacris robertsi*	5	0.68	7.79	3.64	2.73	7	0.76	10.45	5.22	4.21
*Poekilocerus bufonius* ^a^	5	1.31	13.41	7.83	7.04	4	1.74	17.75	12.17	10.93
*Poekilocerus pictus* ^a^	11	1.66	18.33	10.24	8.09	10	2.01	27.08	15.43	13.2
*Prosphena scudderi*	13	0.99	11.7	5.46	6.13	7.00	1.00	14.51	7.03	9.26
*Psedna nana*	1	0.31	4.45	0.64	1.04	1.00	0.37	7.05	2.15	1.62
*Pyrgomorpha conica* ^a^	6	0.71	8.11	2.97	2.72	13	0.69	11.24	4.56	4.76
*Pyrgotettix pueblensis*	6	0.83	8.77	3.48	3.23	7	0.93	11.48	4.92	4.10
*Sphenacris crassicornis*	24	0.84	8.40	3.17	2.90	16	1.05	11.22	5.19	4.88
*Sphenarium histrio*	35	1.31	12.97	5.74	6.39	36	1.05	14.19	7.10	9.10
*Sphenarium planum*	12	1.11	10.69	4.77	5.87	12	0.96	12.33	6.66	9.08
*Sphenarium purpurascens*	2170	1.30	12.62	5.54	6.64	1797	0.85	12.07	6.02	7.94
*Sphenotettix nobilis*	13	0.75	8.23	2.95	2.67	4	0.73	10.58	4.33	4.04
*Tagasta indica* ^a^	10	1.05	13.54	5.53	4.70	6	1.06	16.27	7.82	6.70
*Yunnanites coriacea*	4	1.66	17.99	7.19	8.31	3	1.53	19.89	9.11	10.47
*Zonocerus elegans*	13	1.54	15.64	7.39	7.02	16	1.40	18.51	9.53	9.36
*Zonocerus variegatus*	12	1.67	18.46	8.26	7.39	12	1.45	19.07	9.82	8.44

*Note*: All the measurements are in mm.

Abbreviations: FI, Femur I length; FIII, Femur III length; *n*, sampled size; TL, thorax length; TW, thorax width.

^a^ Winged species.

**TABLE 2:** Lovich and Gibbons (1992) sexual size dimorphism index (SSD) of the four morphological traits measured to males and females of 32 Pyrgomorphid species.

Species	Sexual dimorphism index			
	SSD F1	SSD FIII	SSD TL	SSD TW
*Algete brunneri*	0.36	0.37	0.53	0.70
*Atractomorpha sinensis*	0.16	0.39	0.48	0.63
*Aularches miliaris*	−0.08	0.00	0.09	0.12
*Chrotogonus hemipterus*	0.37	0.56	0.75	0.99
*Colemania sphenarioides*	−0.09	0.25	0.50	0.31
*Dictyophorus griseus*	−0.02	0.11	0.07	0.09
*Dictyophorus spumans*	0.13	0.14	0.24	0.27
*Ichthyotettix mexicanus*	0.05	0.29	0.63	0.52
*Jaragua oviedensis*	0.32	0.20	0.58	0.67
*Mekongiella kingdoni*	−0.25	0.02	0.01	0.03
*Monistria consobrina*	0.12	0.39	0.69	0.87
*Monistria discrepans*	0.21	0.34	0.66	0.67
*Omura congrua*	0.24	0.36	0.58	0.97
*Parapetasia femorata*	0.23	0.49	0.46	0.61
*Phymateus morbillosus*	0.19	0.05	0.31	0.43
*Phymateus viridipes*	0.21	0.25	0.36	0.42
*Piscacris robertsi*	0.10	0.34	0.44	0.54
*Poekilocerus bufonius*	0.33	0.32	0.55	0.55
*Poekilocerus pictus*	0.21	0.48	0.51	0.63
*Prosphena scudderi*	0.01	0.24	0.29	0.51
*Psedna nana*	0.19	0.58	2.36	0.56
*Pyrgomorpha conica*	−0.02	0.39	0.54	0.75
*Pyrgotettix pueblensis*	0.12	0.31	0.41	0.27
*Sphenacris crassicornis*	0.24	0.34	0.64	0.68
*Sphenarium histrio*	−0.20	0.09	0.24	0.42
*Sphenarium planum*	−0.14	0.15	0.40	0.55
*Sphenarium purpurascens*	−0.35	−0.04	0.09	0.20
*Sphenotettix nobilis*	−0.03	0.29	0.47	0.51
*Tagasta indica*	0.01	0.20	0.42	0.42
*Yunnanites coriacea*	−0.08	0.11	0.27	0.26
*Zonocerus elegans*	−0.09	0.18	0.29	0.33
*Zonocerus variegatus*	−0.13	0.03	0.19	0.14

*Note*: Negative values indicated SSD bias in males and positive values indicated SSD bias in females.

Abbreviations: F1, Femur 1; FIII, Femur III length; TL, thorax length; TW, thorax width.

### 2.2 Climatic information

Because temperature and food availability can impact adult body size of grasshoppers, our study incorporated climatic parameters from the sampling localities of each specimen. Geographic coordinates were obtained using Google Earth 7.1.5 (Google Inc 2014), while BioClim provided 14 climatic parameters related to temperature and precipitation (Hijmans et al., 2005). Due to the spatial resolution of the climatic data (30 s = ~1 km^2^) and the scale of this study, we considered that the error associated to the sampling localities is negligible. Precipitation parameters were used as indicators of food availability, as supported by previous studies (Branson, 2010; Cueva del Castillo et al., 2015; Yom‐Tov & Geffen, 2006). Our analysis focused on six climatic parameters directly associated with the growth and reproductive cycles of pyrgomorphids: the annual mean temperature, temperatures seasonality, the temperature of the wettest quarter of the year, annual precipitation, seasonal precipitation, and precipitation of wettest quarter of the year (Table 3). Due to the high correlation among climatic variables and the seasonal presence of nymphal and adult instar of these organisms, this approach allowed us to effectively capture the essential factors impacting their development and reprodcutive patterns.

**TABLE 3:** Climatic parameters estimated for 32 Pyrgomorphid species Bio 1: annual mean temperature°C, Bio 4: temperatures seasonality, Bio 8: temperature of the wettest quarter of the year, Bio 12: annual precipitation, Bio 15: precipitation seasonality, Bio 16: precipitation of wettest quarter of the year.

*Species*	Bio_1	Bio_4	Bio_8	Bio_12	Bio_15	Bio_16
*Algete brunneri*	22.00	126.36	21.00	1096.73	73.01	537.91
*Atractomorpha sinensis*	19.54	717.84	24.16	1326.96	56.85	577.04
*Aularches miliaris*	23.50	164.40	23.68	1433.96	75.82	712.88
*Chrotogonus hemipterus*	22.78	207.64	24.45	534.14	100.27	282.43
*Colemania sphenarioides*	26.12	228.91	26.53	674.25	91.33	333.25
*Dictyophorus griseus*	21.51	126.46	22.11	1156.42	68.54	541.71
*Dictyophorus spumans*	17.36	365.65	19.12	641.38	67.82	305.00
*Ichthyotettix mexicanus*	17.32	229.40	18.52	462.08	78.98	223.63
*Jaragua oviedensis*	28.78	502.62	32.38	95.00	126.02	77.00
*Mekongiella kingdoni*	4.53	665.77	12.25	343.00	129.06	256.00
*Monistria consobrina*	21.67	627.00	28.05	279.00	45.86	110.00
*Monistria discrepans*	17.60	369.58	21.81	1276.60	25.30	420.00
*Omura congrua*	26.25	48.68	25.96	1780.13	49.24	730.82
*Parapetasia femorata*	24.09	73.75	23.72	1804.80	60.00	728.40
*Phymateus morbillosus*	17.93	468.69	20.27	273.39	53.58	126.43
*Phymateus viridipes*	18.33	296.20	20.83	706.68	93.12	390.16
*Piscacris robertsi*	19.11	150.40	19.67	1194.00	101.30	719.00
*Poekilocerus bufonius*	19.04	627.39	10.98	51.00	82.08	28.00
*Poekilocerus pictus*	27.36	345.08	28.11	821.85	88.97	474.70
*Prosphena scudderi*	23.77	113.05	23.96	1839.63	77.32	817.00
*Psedna nana*	15.54	447.68	10.33	460.00	52.93	199.00
*Pyrgomorpha conica*	18.10	565.32	15.53	409.42	71.30	192.26
*Pyrgotettix pueblensis*	15.45	169.92	16.93	474.46	89.18	241.00
*Sphenacris crassicornis*	20.39	322.39	23.01	927.45	85.45	501.75
*Sphenarium histrio*	23.18	0.50	24.01	1003.09	101.79	573.23
*Sphenarium planum*	17.21	0.66	18.93	495.06	88.44	245.00
*Sphenarium purpurascens*	17.07	0.70	18.47	723.13	94.21	414.48
*Sphenotettix nobilis*	16.65	204.56	17.84	1171.29	80.09	600.65
*Tagasta indica*	22.37	403.52	26.33	1935.88	93.58	1147.94
*Yunnanites coriacea*	17.92	429.51	22.35	1289.00	83.28	713.00
*Zonocerus elegans*	23.18	180.53	24.79	901.17	98.64	477.90
*Zonocerus variegatus*	24.14	107.46	24.10	1716.63	61.17	683.54

*Note*: precipitation, Mm; temperature, °C.

### 2.3 Comparative analyses

#### 2.3.1 Phylogenetic reconstruction

To consider the phylogenetic effects in our comparative analysis (see below), we first reconstructed a phylogeny of Pyrgomorphidae based on mitochondrial genome sequences available on GenBank (Benson, 2017) that were generated by previous studies (see Table 4 for accession numbers and references of the used sequences). Our taxon sampling included three outgroups and 32 ingroup species. We aligned all protein‐coding genes individually considering the conservation of reading frames by first translating into amino acids in MUSCLE (Edgar, 2004) using default parameters in Geneious Prime 2023.1. Two ribosomal RNA genes (12S, 16S) were aligned in MAFFT using the E‐INS‐i setting, also in Geneious. Using SequenceMatrix (Vaidya et al., 2011) we concatenated all these individual alignments into a single matrix. We divided this matrix into a total of 15 data blocks (13 protein‐coding and two ribosomal RNA). We performed a maximum likelihood (ML) analysis on the total evidence dataset (14,335 aligned bp and 44 taxa) applying the GTRCAT model to each partition and using RAxML 7.2.8 (Stamatakis et al., 2008) on XSEDE (Extreme Science and Engineering Discovery Environment, https://www.xsede.org) through CIPRES Science Gateway (Miller et al., 2011). Nodal support was evaluated using 1000 replications of rapid bootstrapping implemented in RAxML.

**TABLE 4:** Taxonomic information and Genbank accession numbers for the 35 taxa used in the phylogenetic analysis.

Family	Species	Genbank accession numbers	References
Pyrgomorphidae	*Algete brunneri*	MK514109	Mariño‐Pérez and Song (2019)
Pyrgomorphidae	*Atractomorpha sinensis*	NC011824	Ding et al. (2007)
Pyrgomorphidae	*Aularches miliaris*	MT011442, MT011487, MT011535, MT011581, MT011627, MT011669, MT011715, MT011756, MT011803, MT011845, MT011891, MT011933, MT011973	Song et al. (2020)
Pyrgomorphidae	*Chrotogonus hemipterus*	MK514108	Mariño‐Pérez and Song (2019)
Pyrgomorphidae	*Colemania sphenarioides*	MK531234–MK531254	Mariño‐Pérez and Song (2019)
Pyrgomorphidae	*Dictyophorous spumans*	MK514106	Mariño‐Pérez and Song (2019)
Pyrgomorphidae	*Dictyophorus griseus*	MT011491, MT011538, MT011585, MT011673, MT011719, MT011760, MT011849, MT011895, MT011937, MT011977	Song et al. (2020)
Pyrgomorphidae	*Ichthyotettix mexicanus*	MK531214‐MK531233	Mariño‐Pérez and Song (2019)
Pyrgomorphidae	*Jaragua oviedensis*	MK514195	Mariño‐Pérez and Song (2019)
Pyrgomorphidae	*Mekongiella kingoni*	NC_023921	Zhi et al. (2016)
Pyrgomorphidae	*Monistria consobrina*	MT011427, MT011472,MT011518,MT011564,MT011612,MT011699,MT011741, MT011788, MT011831, MT011877, MT011920	Song et al. (2020)
Pyrgomorphidae	*Monistria discrepans*	MK514105	Mariño‐Pérez and Song (2019)
Pyrgomorphidae	*Omura congura*	MT011429, MT011474, MT011521, MT011567, MT011614, MT011701, MT011743, MT011791	Song et al. (2020)
Pyrgomorphidae	*Parapetasia femorata*	MT011475, MT011522, MT011568, MT011702, MT011744	Song et al. (2020)
Pyrgomorphidae	*Phymateus morbillosus*	MK514103	Mariño‐Pérez and Song (2019)
Pyrgomorphidae	*Phymateus viridipes*	MT011451, MT011497, MT011543, MT011591, MT011636, MT011677, MT011725, MT011766, MT011811, MT011855, MT011901, MT011939, MT011981	Song et al. (2020)
Pyrgomorphidae	*Piscacris robertsi*	MK514096	Mariño‐Pérez and Song (2019)
Pyrgomorphidae	*Poekilocerus bufonius*	MK514102	Mariño‐Pérez and Song (2019)
Pyrgomorphidae	*Poekilocerus pictus*	MT011428, MT011473, MT011520, MT011566, MT011613, MT011657, MT011700, MT011742, MT011790, MT011832, MT011878, MT011960	Song et al. (2020)
Pyrgomorphidae	*Prosphena scudderi*	MK514101	Mariño‐Pérez and Song (2019)
Pyrgomorphidae	*Psedna nana*	MK514100	Mariño‐Pérez and Song (2019)
Pyrgomorphidae	*Pyrgomorpha conica*	Z97616, Z97600, KM384875, KM384853, JF932467, EU031779, EU031778, EU031777, EU031776	Flook et al. (1999); Chapco and Contreras (2011); Fries et al. (2007)
Pyrgomorphidae	*Pyrgotettix pueblensis*	MK531140‐MK531144	Mariño‐Pérez and Song (2019)
Pyrgomorphidae	*Sphenacris crassicornis*	MK514099	Mariño‐Pérez and Song (2019)
Pyrgomorphidae	*Sphenarium histrio*	KU146941	Sanabria‐Urbán et al. (2015)
Pyrgomorphidae	*Sphenarium planum*	KU146980	Sanabria‐Urbán et al. (2015)
Pyrgomorphidae	*Sphenarium purpurascens*	MK514107	Mariño‐Pérez and Song (2019)
Pyrgomorphidae	*Sphenotettix nobilis*	MK514098	Mariño‐Pérez and Song (2019)
Pyrgomorphidae	*Tagasta indica*	MK080200	Mariño‐Pérez and Song (2019)
Pyrgomorphidae	*Yunnanites coriacea*	JQ301463, JQ283277, GQ421456, DQ365908, JQ065110	Lv and Huang (2012); Zhang et al. (2011); and Huo et al. (2007)
Pyrgomorphidae	*Zonocerus elegans*	MT011452, MT011498, MT011544, MT011592, MT011637, MT011678, MT011726, MT011767, MT011812, MT011856, MT011902, MT011940	Song et al. (2020)
Pyrgomorphidae	*Zonocerus variegatus*	MT011449, MT011495, MT011541, MT011589, MT011634, MT011675, MT011723, MT011764, MT011809, MT011853, MT011899, MT011979	Song et al. (2020)
Outgroups			
Acrididae	*Locusta migratoria*	NC_001712	Flook et al. (1995)
Lentulidae	*Lentula callani*	NC_020774	Leavitt et al. (2013)
Pamphagidae	*Prionotropis hystrix*	JX913764	Leavitt et al. (2013)

*Note*: We also provide the authors of the genetic sequences used in this study.

To estimate timing and rates of divergence in the phylogeny of Pyrgomorphidae, we performed a divergence time analysis using BEAST v.2.7.4 (Drummond et al., 2012). So far, there is no reliable fossil calibration point for the family. Therefore, we used the estimated age of the Pyrgomorphidae (109.35 mya) from Song et al. (2020) who used 11 fossil calibration points for inferring the divergence time estimate of Orthoptera. For our divergence time estimate analysis we used the best‐fit models of nucleotide substitution and partitioning scheme recommended by PartitionFinder (Lanfear et al., 2016). Moreover, we relaxed the clock log‐normal model for the clock model, the birth–death model with a uniform distribution as a tree prior, and a log‐normal distribution as a distribution prior for the calibration point. We conducted two separate analyses each for 10 million generations, sampling every 5000 generations. To inspect convergence across independent runs, we used Tracer (Rambaut & Drummond, 2003–2009) and discarded 25% of each run as burn‐in, and combined the two best trees that converged using LogCombiner (Rambaut & Drummond, 2002a). A maximum clade credibility tree was summarized in TreeAnnotator (Rambaut & Drummond, 2002b), and visualized in FigTree.

#### 2.3.2 Body size ancestral state reconstruction

We conducted ancestral character state reconstructions to examine the evolutionary patterns of body size and SSD. We utilized the fastAnc function from the “Phytools” package in R 4.0.1 (R Core Team, 2015), as outlined by Revell (2013). This function estimates the ML value of a continuous trait for internal nodes and then interpolates the states along the branches of the phylogenetic tree. Additionally, we employed the contMap function in “Phytools” to visually represent the ancestral state reconstructions on the phylogeny for the traits under investigation.

#### 2.3.3 Climatic conditions and body size

We conducted PGLS regression models using R packages “nlme” (Pinheiro et al., 2019) and “caper” (Orme et al., 2013) to examine the influence of climatic variables on male and female body size and SSD. We employed corBrownian and gls functions to convert phylogeny into correlation structure object and fit linear models, respectively. Regressed each morphological trait (8 response variables) and SSDi values (4 response variables) individually on six climatic parameters. Utilized stepAIC function from “MASS” package (Venables & Ripley, 2002) to select covariables based on AIC. Models with lower AIC values are deemed superior. We calculated Pagel's lambda using “phytools” package to assess phylogenetic signal of traits. Pagel's lambda ranges from 0 to 1, indicating independence of trait evolution from phylogeny (*λ* = 0), complete adherence to Brownian motion (*λ* = 1), or deviation from expected phylogenetic effect (*λ* < 1) (Pagel, 1999).

#### 2.3.4 Body size differences between winged and wingless grasshoppers

We conducted phylogenetic ANOVA using the ‘geiger’ R package to explore potential differences between winged and wingless males and females. This package, as described by Pennell et al. (2014), incorporates a phylogenetic context by calculating the test statistic for ANOVA and simulating new sets of dependent variables on the phylogenetic tree. The null distribution of the test statistic is obtained under a Brownian‐motion model, following the approach outlined by Garland et al. (1992). Males and females were analyzed independently. To estimate the phylogenetic signal of wing condition (winged or wingless) on the studied species we used the *δ* statistic (Borges et al., 2019), which is a real number that increases when a character evolves following the phylogeny and decreases when it evolves independently. For the estimation of the *δ* statistic we use the “ape” package (Paradis & Schliep, 2019) and the function delta with the default settings (Borges et al., 2019) in R. We test the statistical significance of *δ* statistic by generating a probability distribution of 200 random *δ* statistics (representing no phylogenetic signal) to compare the observed *δ* statistic.

#### 2.3.5 Rensch's rule

To assess whether male body size has diverged more than female body size in pyrgomorphid species (Rensch's rule), we employed the phylogenetic independent contrasts method (Felsenstein, 1985) via the “caper” R package (Orme et al., 2013) to account for species' phylogenetic non‐independence (Harvey & Pagel, 1991). To ensure robust parameter estimates, we excluded outliers with studentized residuals exceeding ±3 (Jones & Purvis, 1997). Before proceeding with further analyses, we examined the independence of the standardized contrasts from their estimated nodal values by plotting them using the “caper” plot function (Felsenstein, 1985). Subsequently, we investigated the allometric relationship between log (male) (dependent variable) and log (female) (independent variable) body size, specifically thorax length, and width, femur III length, and femur I width, by fitting four major axis regressions (model II regression; MA, Sokal & Rohlf, 1981) utilizing the phylogenetic independent contrasts (Garland et al., 1992). Rensch's rule predicts the slope of male on female size to be significantly steeper than 1. To account for the expected mean value of contrasts being zero, we enforced the MA regression to pass through the origin (Sol et al., 2014). We reported the major axis regression slopes (*β*), along with their corresponding 95% lower and upper confidence intervals, calculated using the “smatr” R package (Warton et al., 2006, 2012).

## 3 RESULTS

### 3.1 Phylogenetic reconstruction

**FIGURE 3:**
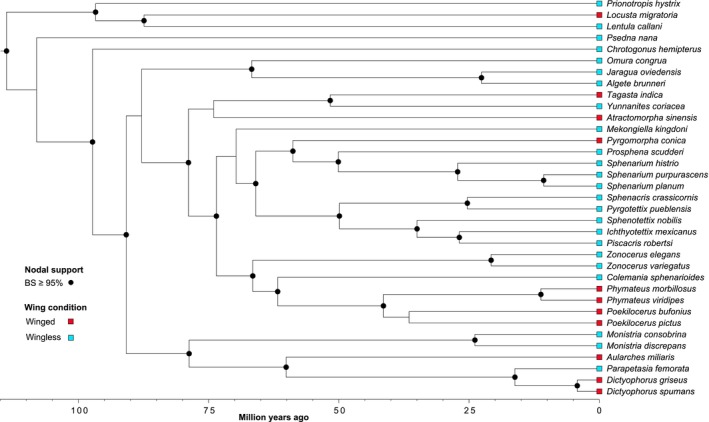
Dated phylogeny of Pyrgomorphidae and wing condition of the included species. The phylogeny is based on maximum likelihood analysis of mitochondrial genome sequences of 35 species (32 Pyrgomorphidae and 3 outgroup species). Black circles on the tree represent nodes with bootstrap support values ≥95%, nodes without black circles had lower support (bootstrap <80%). Colored boxes at the tips indicate wing condition, red for winged and blue for wingless species.

We obtained a fully resolved phylogeny of Pyrgomorphidae (Figure 3), with most phylogenetic relationships highly supported (bootstrap values ≥95%). Our phylogeny is largely consistent with previous phylogenetic studies (Mariño‐Pérez & Song, 2019; Zahid et al., 2021) and phylogenomic inferences (Song et al., 2020) on this group, with minor differences in nodal support values.

### 3.2 Ancestral state reconstruction of body size

**FIGURE 4:**
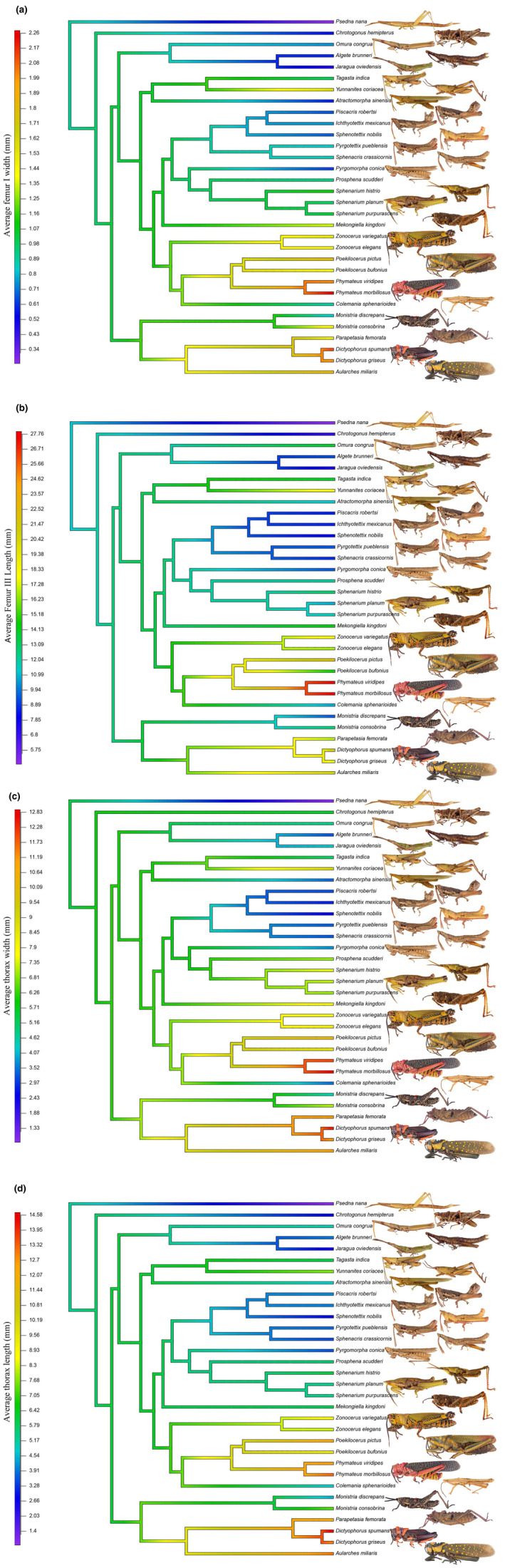
Ancestral phylogenetic reconstructions of (a) femur I width, (b) femur III length, (c) thorax length, and (d) width for 32 pyrgomorphid grasshoppers' taxa performed in R package “phytools” (Revell, 2013). For the analysis, we used the ultrametric phylogeny and the values of SDI estimated for each species.

We obtained morphological information for 32 grasshopper species that were used to perform ancestral state reconstruction of body size. This sample includes species from Africa, America, Australia, and Asia. Based on the current range of pyrgomorphid size variation, the body size reconstruction suggests that the ancestral pyrgomorphid likely had an intermediate size. The femur III and I were smaller than those of the present species, while the thorax length and width were larger than the average in the present species (Table 5). Overall, the group exhibited a trend toward increasing body size. However, there have been at least four independent times where a trend toward evolving a smaller body size has occurred (Figure 4a–d). Approximately, 32% of the nine species analyzed showed a trend toward the reduction of their body size: *Psedna nana, Algete brunneri, Jaragua oviedensis, Piscacris robertsi, Ichthyotettix mexicanus, Sphenotettix nobilis, Pyrgotettix pueblensis, Sphenacris crassicornis*, and *Pyrgomorpha conica*.

**TABLE 5:** Body size differences between the hypothetical ancestor of Pyrgomorphidae and the average size for the femur III length, femur I width, thorax length, and thorax width of the 33 species of the family measured for this study.

	Femur III length	Femur I width	Thorax length	Thorax width
Ancestor	≈11 mm	≈0.85 mm	≈4.65 mm	≈6.00 mm
Present species	14.09 mm	1.17 mm	3.66 mm	3.26 mm

### 3.3 Climatic conditions and body size

According to the comparisons between the pgls models with the AIC criteria, the best predictor model was only associated with the mean annual temperature, and the thorax width SSD (*F*_(3,28)_ = 3.385, *p* = .032). The mean annual temperature showed a positive relationship with the thorax width SSD (see Table 6). Thus, lower annual temperatures tend to favor the presence of species with relatively smaller females, while environments with higher temperatures are more likely to be inhabited by species exhibiting the highest sexual size dimorphism (SSD) bias toward females. Moreover, the divergence between the sexes is not constrained by the phylogenetic history of the group (*λ* = .66; *p* = .18).

**TABLE 6:** (a) Parameters included in the significative multiple regression model of the SSDi of the thorax width of pyrgomorph grasshoppers on the climatic variables and (b) Delta Akaike Information for different regression models used to explain variation in the thorax width SSD in pyrgomorphid grasshoppers as a function of climatic variables.

(a)				
	Estimate	SE	*t*	*p*
Intercept	0.203	0.264	0.7672	.449
A	0.0236	0.009	2.646	**.013**
B	0.0003	0.0002	1.552	.131
E	−0.003	0.002	−1.722	.096

(b)			
Model	AIC	∆AIC	*r* ^2^
A + B + C + D + E + F	11.43	5.54	.29
A + B + C + D + E	9.46	3.57	.28
A + B + C + E	7.66	1.77	.27
A + B + E	5.9	0.01	.26
A	5.89	0.00	.19

*Note*: Results in bold represent the best regression model as indicated by ∆ AIC. SE, *t* and *p*‐values are shown.

Abbreviations: ∆AIC, delta Akaike information criterion; A, annual mean temperature; AIC, Akaike's information criterion; B, temperature seasonality; C, mean temperature of the wettest quarter of the year; D, annual precipitation; E, precipitation seasonality; F, precipitation of the wettest quarter of the year; *r* ^2^, variance explained by each model; SE, standard error.

### 3.4 Body size differences between winged and wingless grasshoppers

**FIGURE 5:**
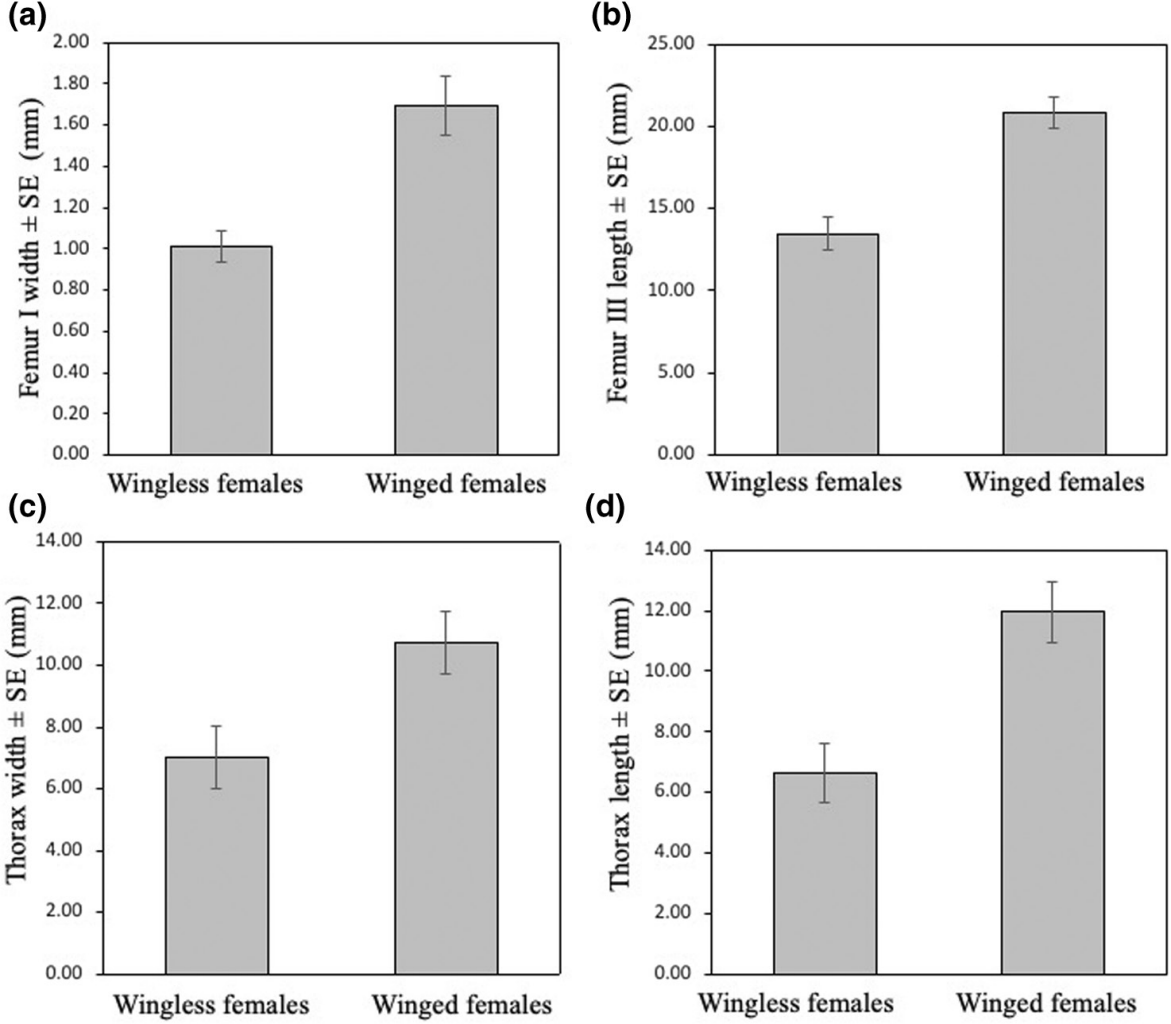
Mean and standard error values for (a) femur I width, (b) femur III length, (c) thorax width and (d) thorax length of the wingless and winged females of the family Pyrgomorphidae.

**FIGURE 6:**
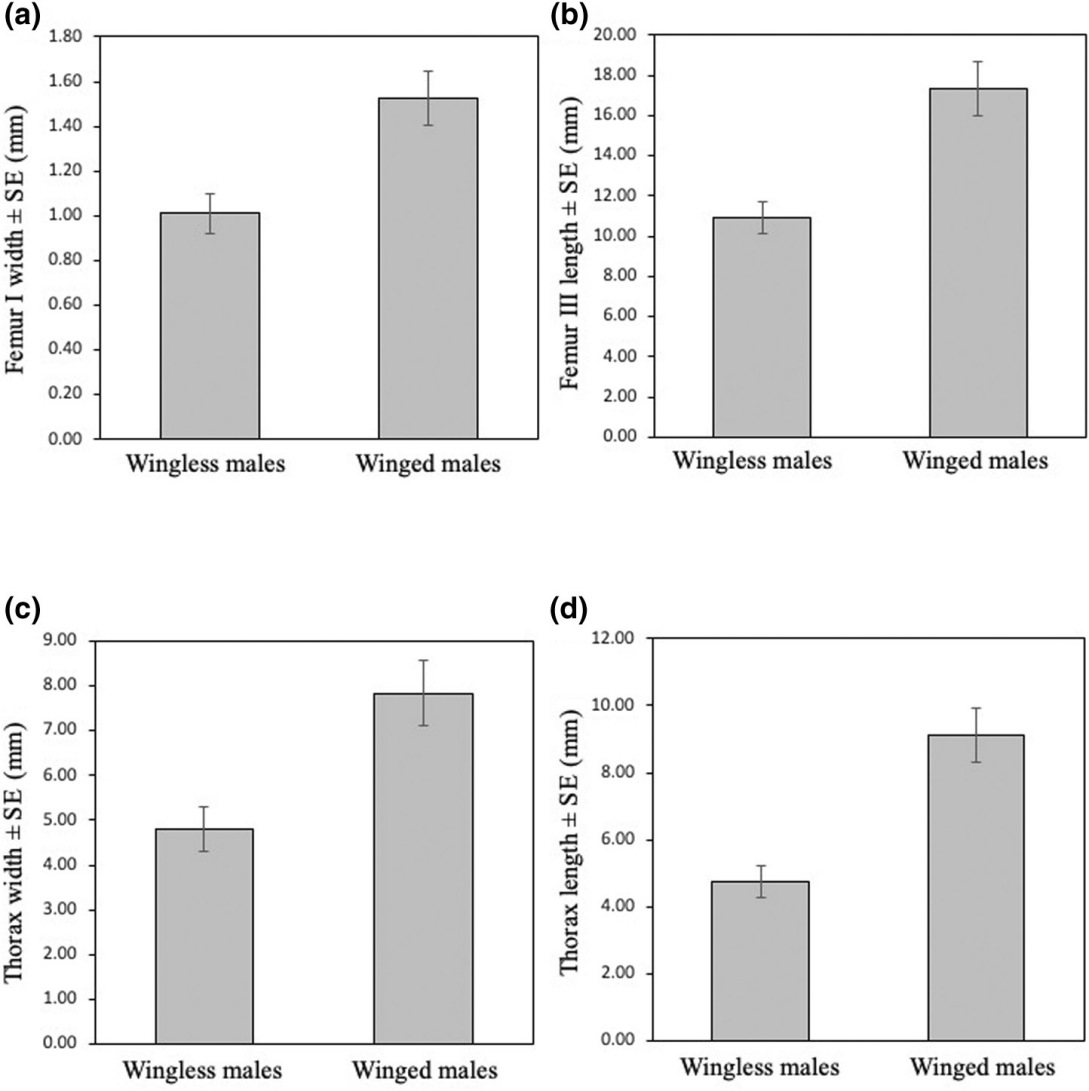
Mean and standard error values for (a) femur I width, (b) femur III length, (c) thorax width, and (d) thorax length of the wingless and winged males of the family Pyrgomorphidae.

We found specimens for 10 winged and 22 wingless species. All the branches of the phylogeny are represented by winged and wingless species (Figure 3), suggesting that throughout the evolutionary history of pyrgomorphids, the loss of wings has been recurrent. There were significant body size differences between wingless and winged males and females. However, opposite to our hypothesis winged males and females were larger than males and females of wingless species (Figures 5 and 6). After controlling for phylogenetic effects, just the femur III length and the thorax length of the males remain significant (Table 7), while for the females remain significant the F I, thorax with and the femur III length (Table 8). Moreover, we found a weak, but statisctically significant phylogenetic signal for wing condition on the family (*δ* = .952, *p*‐value = .02), which is consistent with the scattered distribution of the winged condition across the phylogeny with few clades mainly comprising winged species (Figure 3).

**TABLE 7:** Phylogenetic analyses of variance for (a) femur III length, (b) femur I width, (c) thorax length, and (d) thorax width of wingless and winged males of the family Pyrgomorphidae.

Source	df	SS	MS	*F*	*p*	*p* *
a. Femur III length						
Wing condition	1	0.273	0.273	11.118	**.002**	**.009**
Error	30	0.737	0.025			
b. Femur I width						
Wing condition	1	0.229	0.229	5.968	**.020**	.079
Error	30	1.152	0.038			
c. Thorax length						
Wing condition	1	0.595	0.595	10.230	**.003**	**.018**
Error	30	1.745	0.058			
d. Thorax width						
Wing condition	1	0.318	0.318	6.038	**.020**	.062
Error	30	1.582	0.053			

Abbreviations: df, degrees of freedom; *F*, *F* values; MS, mean squares, *p*, *p*‐value; SS, sum of squares.

* *p*‐value corrected by the phylogeny (p*) are shown.

**TABLE 8:** Phylogenetic analyses of variance for (a) femur III length, (b) femur I width, (c) thorax length, and (d) thorax width of wingless and winged females of the family Pyrgomorphidae.

Source	df	SS	MS	*F*	*p*	*p* *
a. Femur III length						
Wing condition	1	0.240	0.240	15.030	**.0005**	**.005**
Error	30	0.478	0.016			
b. Femur I width						
Wing condition	1	0.300	0.300	10.516	**.003**	**.025**
Error	30	8.858	0.028			
c. Thorax length						
Wing condition	1	0.447	0.447	14.077	**.0008**	**.004**
Error	30	0.953	0.032			
d. Thorax width						
Wing condition	1	0.256	0.256	6.259	**.018**	.083
Error	30	1.228	0.041			

Abbreviations: df, degrees of freedom; *F*, *F* values; MS, mean squares; *p*, *p*‐value; SS, sum of squares.

* *p*‐value corrected by the phylogeny (p*) are shown.

### 3.5 Rensch's rule

**FIGURE 7:**
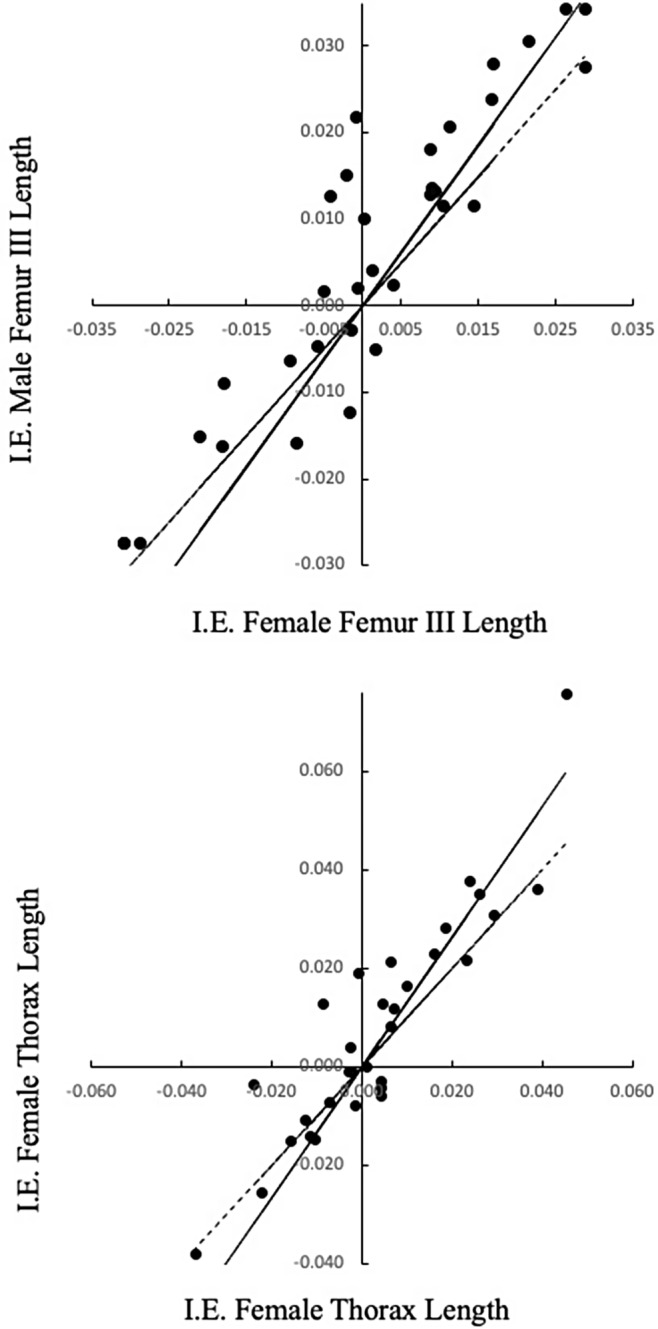
Significant allometric major axis regressions of independent contrasts (IC) of (a) the femur III length of males on the femur III length of females', and (b) the thorax length of the males on the thorax length of the females. Dashed lines indicate isometry (*β* = 1).

Our results indicate strong coevolution between males and females (Table 9). Males' traits showed slopes greater than 1.0 when regressed on females' traits, with femur III and thorax lengths significantly differing from isometry (*β* = 1.0; Figure 7).

**TABLE 9:** Mayor axis regressions of the independent contrast of male traits on the female traits, slope values (*β*) lower and upper confident intervals, variance explained by the models and *p*‐values of the regression, and the deviation of *β* = 1 (*H*_0_) are shown.

Regresion	*β*	*β* lower limit	*β* upper limit	*r* ^2^	*p*‐Value regresion	*H*_0_: *β* = 1 *p*
FI M on FI F	1.15	0.94	1.41	.77	<.0001	.178
F III M on F III F	1.24	1.00	1.53	.77	<.0001	**.045**
TL M on TL F	1.32	1.11	1.59	.81	<.0001	**.003**
TW M on TW F	1.10	0.91	1.36	.77	<.0001	.333

*Note*: In all the cases degrees of freedom (df) = 29.

Abbreviations: F III F, Femur III females; F III M, Femur III males; FI F, Femur I females; FI M, Femur I males; TL F, thorax length females; TL M, thorax length males; TW F, thorax width females; TW M, thorax width males.

## 4 DISCUSSION

Considering the current variation in body size of the members of the family Pyrgomorphidae, our results suggest that these grasshoppers have evolved from an ancestor of intermedium size. The absence of phylogenetic constraints (*λ* = .66; *p* = .18) has not limited the diversification of the body in this linage of grasshoppers, allows for a greater flexibility in adapting to mean annual temperatures, which may contribute to the observed differences in body size between males and females. Furthermore, the disparity in wing development in pyrgomorphs is closely linked to variations in body size. This correlation suggests that the evolution of wings in these insects is intricately tied to their physical dimensions. As body size differs among pyrgomorph species, so too does the development of their wings. This phenomenon underscores the significance of body size in shaping the morphological characteristics of these insects. The insight into the connection between body size and wing development sheds light on the adaptive strategies employed by pyrgomorphs and provides valuable information for further research into the evolutionary mechanisms governing insect morphology.

Female fecundity and male mating success tend to increase with body size, but the optimal body size often differs between males and females, generating sexual size dimorphism (SSD). The differences between females and males are further influenced by climatic conditions such as temperature and season length, which affect growth rates and development times. Seasonality plays a crucial role in determining the duration of optimal developmental conditions for both males and females. As a result, the body size differences between males and females within pyrgomorph species are not only shaped by reproductive strategies, but also by environmental factors that regulate growth and development.

### 4.1 Ancestral state reconstruction of body size

The body size reconstruction suggests that the members of the family Pyrgomorphidae have evolved from an ancestor of the intermedium size. During the evolution of the groups, there have been trends to increase and decrease body size. Both trends could be modeled by several selective pressures associated with the environments that occupy the species of the family. Small body size often correlates with a high seasonal environment, where limited time is available to complete the life cycle. Thus, members of the subtribe Sphenariina (Genus *Jaragua*, *Prosphena*, and S*phenarium*) showed a trend to decrease body size. *Piscacris robertsi*, *Ichthyotettix mexicanus*, *Sphenotettix nobilis*, *Pyrgotettix pueblensis*, and *Sphenacris crassicornis* are phylogenetical related and have evolved at the highlands of central Mexico, which are highly seasonal (Sanabria‐Urbán et al., 2015). *Psedna nana* occurs in the western side of Australia and may be evolved also in a highly seasonal environment, whereas members of the tribes Phymateini (Genus *Phymateus* and *Zonocerus*) and Poekilocerini (*Poekilocerus*) tend to increase and decrease body size. Interestingly, *Pyrgomorpha conica* is the only small species with wings represented in the phylogeny, perhaps its wide distribution across South Europe, North Africa, and Central Asia may be attributed to its high dispersion capacity associated with flight. In any case, we must point out that the limited number of species considered in this phylogenetic reconstruction could limit the accurate interetation of the ancestral state of the lineage and its evolutionary trends.

### 4.2 Climatic conditions and body size

The impact of natural and sexual selection on the body size divergence between males and females of pyrgomorphids appears to vary across different environmental conditions. Interestingly, our findings indicate that it is the mean annual temperature rather than precipitation that plays a significant role in this divergence. We observed that species with less sexual dimorphism tend to inhabit cooler environments, whereas those with a greater bias toward females are found in warmer regions. These patterns suggest a strong relationship between maturation time and body size in these grasshoppers (Cueva del Castillo & Nuñez‐Farfán, 1999, 2002). As body size is strongly correlated with maturation time in insects, faster sexual maturation is reached at the expense of having a small body size (Thornhill & Alcock, 1983). However, in Orthoptera and Coleoptera there are species in which the instar number is higher in favorable conditions. (Esperk, Tammaru, & Nylin, 2007; Esperk, Tammaru, Nylin, & Teder, 2007). Interestinlgly, *Sphenarium histrio*, a species represented in this study, male and female collected from low altitude sites in the field and reared in the laboratory were larger than those from a high altitude. Grasshoppers from a high altitude hatched earlier, had a shorter development time, presented fewer instars, and were smaller than grasshoppers from the other sampling sites (Ramírez‐Delgado & Cueva del Castillo, 2022). This local adaptation highlights the intricate interplay between environmental factors and the evolution of sexual dimorphism in these insects. However, in the absence of phylogenetic constraints, when fecundity selection outweighs sexual selection, it is likely that larger female body sizes will result compared with males.

### 4.3 Body size differences between winged and wingless grasshoppers

Opposite to our hypotheses, winged males and females were larger than wingless species, suggesting that the costs to produce and maintain the wings do not impact the resources canalized to body size. In fact, it is possible that if there are these costs for the winged species, maybe they are compensated for an increase in the resources canalized to body size. Alternatively, given the toxicity of some of the largest species (Yang et al., 2019), lower predation levels may lead to longer life spans and larger body sizes, indirectly benefiting fecundity. This effect could be more pronounced in warmer and less seasonal environments, where natural selection favors these traits (Horne et al., 2015).

### 4.4 Rensch's rule

Despite the potential tradeoffs between maturation time and body, and the size differences between winged and wingless grasshoppers, positive directional sexual selection on femur III and thorax lengths may provide insight into the evolution of SSD in Pyrgomorphidae, with male size hyperallometry reflecting their greater evolutionary divergence due to strong mate competition. Larger males have advantages in accessing females and resisting takeover attempts (Cueva del Castillo et al., 1999; Cueva del Castillo & Nuñez‐Farfán, 1999; Wickler & Seibt, 1985), with higher mating success and extraordinary copulation and mate‐guarding durations reported in some species (Cueva del Castillo, 2003; Descamps M & Wintrebert D., 1966; Wickler & Seibt, 1985). These behaviors may decrease the possibility of sperm competition.

The family Pyrgomorphidae shows great diversification, successfully colonizing a variety of niches across their distribution range from Asia to Tropical America (Sanabria & Cueva del Castillo, 2020). This broad range of habitats suggests that the relative impact of natural and sexual selection on body size has likely shifted in response to changing environmental conditions, that favored the body size divergence of females and males. Variations in pyrgomorphids may be closely linked to their evolution in distinct continents. For example, it is noteworthy that toxicity is absent in numerous neotropical species of the Genus *Sphenarium* and *Prosphena*. This suggests that environmental factors and geographical isolation have played a significant role in shaping the characteristics of these species. Understanding these differences can provide valuable insights into the intricate processes of evolution and adaptation. The specific causal mechanisms driving these changes in SSD remain an open question that requires further investigation. In any case, further investigation is required to determine the causal mechanisms behind the changes in the direction of SSD in this group of grasshoppers.

## AUTHOR CONTRIBUTIONS

**Raúl Cueva del Castillo:** Conceptualization (equal); data curation (lead); formal analysis (equal); funding acquisition (lead); investigation (lead); methodology (lead); project administration (lead); resources (equal); software (equal); supervision (lead); validation (equal); visualization (lead); writing – original draft (lead); writing – review and editing (equal). **Salomón Sanabria‐Urbán:** Data curation (equal); formal analysis (equal); investigation (equal); methodology (equal); software (equal); validation (equal). **Ricardo Mariño‐Pérez:** Data curation (equal); investigation (equal); validation (equal); writing – review and editing (equal). **Hojun Song:** Data curation (equal); formal analysis (equal); methodology (equal); software (equal); supervision (lead); validation (lead); writing – review and editing (equal).

## CONFLICT OF INTEREST STATEMENT

None declared.

## Supporting information


Table S1

## Data Availability

The GenBank accession numbers of the used sequences are shown in Table S1 and the final alignment used and the resulting phylogeny are available at TreeBASE under submission 31,267.
