# Correction to “Annual Temperature, Body Size, and Sexual Size Dimorphism in the Evolution of Pyrgomorphidae”

**DOI:** 10.1002/ece3.70446

**Published:** 2024-10-22

Cueva del Castillo, R., Sanabria‐Urbán, S., Mariño‐Pérez, R., & Song, H. (2024). Annual temperature, body size, and sexual size dimorphism in the evolution of pyrgomorphidae. Ecology and Evolution, 14, e70188. https://doi.org/10.1002/ece3.70188

An incorrect version of Figure 7, which includes inaccurately labeled graphs and axes, was mistakenly included in the final version of the manuscript. We have replaced the erroneous figure with a new one that is free from any mistakes.

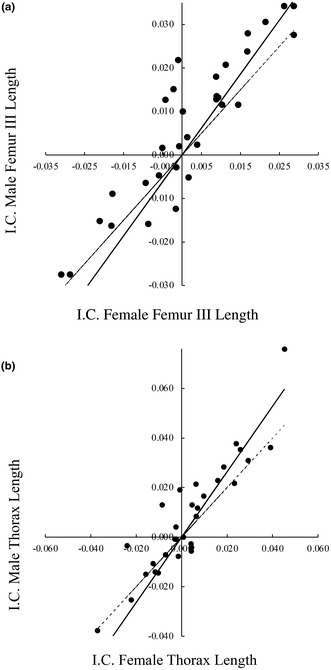

We apologize for this error and any inconvenience this may have caused.

