# Supplementary material for: Annual temperature, body size, and sexual size dimorphism in the evolution of Pyrgomorphidae
Source: Ecol Evol. 2024 Aug 21;14(8):e70188. doi: 10.1002/ece3.70188 (PMC11338691; doi:10.1002/ece3.70188)
Supplement: Supplementary file 1 — Table S1 [file ECE3-14-e70188-s001.docx]

Table S1. Taxa included in the reconstructed phylogeny of Pyrgomorphidae. *Lentula callani, Locusta migratoria* and *Prionotropis hystrix* were incorporated as outgroups*.*

| **Family** | **Subfamily** | **Tribe** | **Species** | **Genbank Accession number** | **Reference** |
| --- | --- | --- | --- | --- | --- |
| Lentulidae |  |  | *Lentula callani* | NC_020774 | Leavitt et al. (2013) |
| Acrididae |  |  | *Locusta migratoria* | NC_001712 | Flook et al. (1995) |
| Pamphagidae |  |  | *Prionotropis hystrix* | JX913764 | Leavitt et al. (2013) |
| Pyrgomorphidae | Orthacridinae | Ichthiacridini | *Ichthiacris rehni* | MK531145-MK531153 | Mariño-Pérez and Song (2019) |
| Pyrgomorphidae | Orthacridinae | Ichthiacridini | *Sphenacris crassicornis* | MK514099 | Mariño-Pérez and Song (2019) |
| Pyrgomorphidae | Orthacridinae | Ichthyotettigini | *Ichthyotettix mexicanus* | MK531214-MK531233 | Mariño-Pérez and Song (2019) |
| Pyrgomorphidae | Orthacridinae | Ichthyotettigini | *Piscacris robertsi* | MK514096 | Mariño-Pérez and Song (2019) |
| Pyrgomorphidae | Orthacridinae | Ichthyotettigini | *Pyrgotettix pueblensis* | MK531140-MK531144 | Mariño-Pérez and Song (2019) |
| Pyrgomorphidae | Orthacridinae | Ichthyotettigini | *Sphenotettix nobilis* | MK514098 | Mariño-Pérez and Song (2019) |
| Pyrgomorphidae | Orthacridinae / | Orthacridini | *Caprorhinus* sp. | MK514097 | Mariño-Pérez and Song (2019) |
| Pyrgomorphidae | Orthacridinae | Popoviini | *Colemania sphenarioides* | MK531234-MK531254 | Mariño-Pérez and Song (2019) |
| Pyrgomorphidae | Orthacridinae | Psednurini | *Psedna nana* | MK514100 | Mariño-Pérez and Song (2019) |
| Pyrgomorphidae | Pyrgomorphinae | Atractomorphini | *Atractomorpha sinensis* | NC011824 | Ding et al. (2007) |
| Pyrgomorphidae | Pyrgomorphinae | Chrotogonini | *Chrotogonus hemipterus* | MK514108 | Mariño-Pérez and Song (2019) |
| Pyrgomorphidae | Pyrgomorphinae | Desmopterini | *Desmoptera irianica* | MK531154-MK531165 | Mariño-Pérez and Song (2019) |
| Pyrgomorphidae | Pyrgomorphinae | Dictyophorini | *Dictyophorus griseus* | MT011491, MT011538, MT011585, MT011673, MT011719, MT011760, MT011849, MT011895, MT011937, MT011977 | Song et al. (2020) |
| Pyrgomorphidae | Pyrgomorphinae | Dictyophorini | *Dictyophorous spumans* | MK514106 | Mariño-Pérez and Song (2019) |
| Pyrgomorphidae | Pyrgomorphinae | Dictyophorini | *Parapetasia femorata* | MT011475, MT011522, MT011568, MT011702, MT011744 | Song et al. (2020) |
| Pyrgomorphidae | Pyrgomorphinae | Monistriini | *Monistria consobrina* | MT011427, MT011472, MT011518, MT011564, MT011612, MT011699, MT011741, MT011788, MT011831, MT011877, MT011920 | Song et al. (2020) |
| Pyrgomorphidae | Pyrgomorphinae | Monistriini | *Monistria discrepans* | MK514105 | Mariño-Pérez and Song (2019) |
| Pyrgomorphidae | Pyrgomorphinae | Omurini | *Algete brunneri* | MK514109 | Mariño-Pérez and Song (2019) |
| Pyrgomorphidae | Pyrgomorphinae | Omurini | *Jaragua oviedensis* | MK514195 | Mariño-Pérez and Song (2019) |
| Pyrgomorphidae | Pyrgomorphinae | Omurini | *Omura congura* | MT011429, MT011474, MT011521, MT011567, MT011614, MT011701, MT011743, MT011791 | Song et al. (2020) |
| Pyrgomorphidae | Pyrgomorphinae | Phymateini | *Phymateus morbillosus* | MK514103 | Mariño-Pérez and Song (2019) |
| Pyrgomorphidae | Pyrgomorphinae | Phymateini | *Phymateus viridipes* | MT011451, MT011497, MT011543, MT011591, MT011636, MT011677, MT011725, MT011766, MT011811, MT011855, MT011901, MT011939, MT011981 | Song et al. (2020) |
| Pyrgomorphidae | Pyrgomorphinae | Phymateini | *Zonocerus variegatus* | MT011449, MT011495, MT011541, MT011589, MT011634, MT011675, MT011723, MT011764, MT011809, MT011853, MT011899, MT011979 | Song et al. (2020) |
| Pyrgomorphidae | Pyrgomorphinae | Phymateini | *Zonocerus elegans* | MT011452, MT011498, MT011544, MT011592, MT011637, MT011678, MT011726, MT011767, MT011812, MT011856, MT011902, MT011940, | Song et al. (2020) |
| Pyrgomorphidae | Pyrgomorphinae | Poekilocerini | *Poekilocerus bufonius* | MK514102 | Mariño-Pérez and Song (2019) |
| Pyrgomorphidae | Pyrgomorphinae | Poekilocerini | *Poekilocerus pictus* | MT011428, MT011473, MT011520, MT011566, MT011613, MT011657, MT011700, MT011742, MT011790, MT011832, MT011878, MT011960 | Song et al. (2020) |
| Pyrgomorphidae | Pyrgomorphinae | Pyrgomorphini | *Ochrophlegma vittifera* | MK514104 | Mariño-Pérez and Song (2019) |
| Pyrgomorphidae | Pyrgomorphinae | Pyrgomorphini | *Pyrgomorpha conica* | Z97616, Z97600, KM384875, KM384853, JF932467, EU031779, EU031778, EU031777, EU031776 | Flook et al. (1999); Ruiz-Ruano et al. (unpublished); Chapco and Contreras (2011); Fries et al. (2007) |
| Pyrgomorphidae | Pyrgomorphinae | Pyrgomorphini | *Stenoscepa* sp. | MT011446, MT011492, MT011586, MT011631, MT011720, MT011761, MT011850, MT011896 | Song et al. (2020) |
| Pyrgomorphidae | Pyrgomorphinae | Pyrgomorphini | *Tanita purpurea* | MK514110 | Mariño-Pérez and Song (2019) |
| Pyrgomorphidae | Pyrgomorphinae | Sphenariini | *Mekongiana xiangchengensis* | NC_014450 | Zhao et al. (2010) |
| Pyrgomorphidae | Pyrgomorphinae | Sphenariini | *Mekongiella kingoni* | NC_023921 | Zhi et al. (2016) |
| Pyrgomorphidae | Pyrgomorphinae | Sphenariini | *Prosphena scudderi* | MK514101 | Mariño-Pérez and Song (2019) |
| Pyrgomorphidae | Pyrgomorphinae | Sphenariini | *Sphenarium purpurascens* | MK514107 | Mariño-Pérez and Song (2019) |
| Pyrgomorphidae | Pyrgomorphinae | Sphenariini | *Sphenarium planum* | KU146980 | Sanabria_Urban et al. (2015) |
| Pyrgomorphidae | Pyrgomorphinae | Sphenariini | *Sphenarium histrio* | KU146941 | Sanabria_Urban et al. (2015) |
| Pyrgomorphidae | Pyrgomorphinae | Sphenariini | *Yunnanites coriacea* | JQ301463, JQ283277, GQ421456, DQ365908, JQ065110 | Lv and Huang (2012); Zhang et al. (2011); Huo et al. (2007); Cui and Huang (unpublished); Bai and Huang (unpublished) |
| Pyrgomorphidae | Pyrgomorphinae | Tagastini | *Tagasta indica* | MK080200 | Mariño-Pérez and Song (2019) |
| Pyrgomorphidae | Pyrgomorphinae | Taphronotini | *Aularches miliaris* | MT011442, MT011487, MT011535, MT011581, MT011627, MT011669, MT011715, MT011756, MT011803, MT011845, MT011891, MT011933, MT011973 | Song et al. (2020) |
